# Comparison of a Tumor-Ratio–Metastasis Staging System and the 8th AJCC TNM Staging System for Gastric Cancer

**DOI:** 10.3389/fonc.2021.595421

**Published:** 2021-07-08

**Authors:** Miaoquan Zhang, Chao Ding, Lin Xu, Biyi Ou, Shoucheng Feng, Guoqiang Wang, Wei Wang, Yao Liang, Yingbo Chen, Zhiwei Zhou, Haibo Qiu

**Affiliations:** ^1^ Department of Gastric Surgery, State Key Laboratory of Oncology in South China, Collaborative Innovation Center for Cancer Medicine, Sun Yat-sen University Cancer Center, Guangzhou, China; ^2^ School of Public Health (Shenzhen), Sun Yat-Sen University, Guangzhou, China; ^3^ Department of Gastrointestinal Surgery, The Second Affiliated Hospital of Guangzhou Medical University, Guangzhou, China

**Keywords:** lymph node ratio, gastric cancer, staging, prognosis, TNM (8th edition)

## Abstract

**Background:**

Despite the implementation of the 8th American Joint Committee on Cancer (AJCC) TNM staging system for gastric cancer (GC) in 2017, it still holds a significant level of stage migration which affects patients’ proper classification and accurate prognosis. Here, to reduce this effect, we evaluated the prognostic value of a lymph node ratio (LNR) and established a novel tumor–ratio–metastasis (TRM) staging system.

**Method:**

The data of 15,206 GC patients from the Sun Yat-sen University Cancer Center (Training set; n=2,032) and the US Surveillance, Epidemiology, and End Results (SEER) database (Validation set; n=13,174) were analyzed. The training set was classified into 5 LNR categories, based on which the novel TRM staging system was constructed. The overall survival (OS) between the TRM and AJCC TNM systems was compared in the training set and validated in the validation set. The likelihood ratio *x*^2^, liner trend *x*^2^, C-index, and Akaike information criterion (AIC) values were used to measure the discriminatory ability between the two different staging systems. Decision curve analyses (DCAs) were conducted to test the clinical value of the two staging systems.

**Result:**

The patients were classified into the following categories: LNR0: 0%, LNR1: 0%<LNR ≤ 10%, LNR2: 10%<LNR ≤ 25%, LNR 3a: 25%<LNR ≤ 60%, and LNR 3b: LNR>60%. Univariate analyses demonstrated that the log-rank *x*^2^ of the LNR stage (Training/Validation set: *x*^2^ = 463.1/2880.8) was larger than the AJCC pN stage (Training/Validation set: *x*^2^ = 281.5/2240.8). For both the training set and validation set, stratified analyses using the Kaplan-Meier method identified significantly heterogeneous OS in every pN category but only one using the LNR. The TRM staging system had higher likelihood ratio *x*^2^, liner trend *x*^2^, C-index and smaller AIC values than the TNM system.

**Conclusion:**

The TRM staging system demonstrated improved homogeneity and discriminatory ability in predicting the prognosis of GC patients compared with the AJCC TNM staging system.

## Introduction

Gastric Cancer (GC) is still the fifth most frequently diagnosed cancer and the third leading cause of cancer deaths worldwide despite declining incidence in the past years (1–3). The overall trend of GC incidence, which is estimated by age-standardizing among the reference population, may cover up the significant age-specific features. Several studies have indicated that reverse trends of incidence were observed among young people in both the West and the East (4–8), leading to greater loss in life-expectancy among younger patients.

Since treatments are directed based on the stage of patients at the time of diagnosis and post-surgery, patients should be accurately staged for optimal treatment. Currently, the most commonly used staging system for GC is the American Joint Committee on Cancer (AJCC) TNM system, which stages patients according to the depth of tumor infiltration (T), number of regional metastatic lymph nodes (N), and status of distant metastasis (M). In the 8th AJCC TNM system, patients with no metastasized LNs, 1-2, 3-6, 7-15 or >15 metastatic LNs are classified as N0, N1, N2, N3a or N3b, respectively. For proper staging, the AJCC recommends at least 16 regional lymph nodes be pathologically assessed. However, the number of resected lymph nodes varies widely, depending on the actual number of regional LNs, surgical technique, surgeons’ skills, and/or pathological procedures. This may lead to stage migration, also called the Will Rogers phenomenon (9, 10); i.e. if the number of retrieved LNs is less than 16, this could lead to down-migration of the N stage.

Lymph node ratio (LNR), also called the node ratio (Nr) or metastatic lymph node ratio (MLNR), is defined as the number of MLN divided by the number of retrieved lymph nodes (11). It has been shown as an effective alternative to decrease the risk of stage migration. Several previous studies have analyzed the prognostic role of LNR and found that LNR is an independent prognostic factor for GC. These studies demonstrated that LNR could better stratify the survival of GC patients than the AJCC pN status (12–18). However, such studies were performed mostly using the 7th or earlier edition of the AJCC TNM system. Since the latest AJCC, the 8th TNM staging system has been released and is superior to 7th edition (19–21). Here, the present study aims to evaluate whether a better N staging system can be provided by replacing the absolute number of regional node metastasis with lymph node ratio, and whether a novel tumor–ratio–metastasis (TRM) staging system shows a better performance compared with the 8th edition AJCC TNM system.

## Methods

### Chinese Cohort and Follow-up

This was a retrospective study comprising of the clinicopathological data of 2,032 GC patients who underwent surgical treatment from January 2000 to June 2017 at the Sun Yat-sen University Cancer Center (SYSUCC; Guangzhou, China). The main eligibility criteria for the study inclusion were histologically confirmed R0 resection. Exclusion criteria were (i) presence of other simultaneous cancer(s) and distant metastasis, (ii) underwent R1 or R2 resection, (iii) had stump or recurrent cancer, (iv) underwent preoperative anti-cancer treatment, (v) died during the perioperative period, and (vi) had incomplete follow-up data. Gastrectomy with lymphadenectomy (D1/D2) was performed by surgeons abiding to the JGCA guidelines, with more than 10 years of experience in performing gastrectomy (22). The data of patients from SYSUCC were used as the training cohort for establishing a hypothetical LNR stage and TRM staging system.

Follow-up assessments including clinical and laboratory examinations were conducted every 3 months for the first 2 years, every 6 months during the 3rd to 5th years, and every 12 months thereafter until death. The primary endpoint was 5-year overall survival (OS). OS was estimated from the date of operation until death or last follow-up contact (February 29, 2020), which was used as a measure to indirectly reflect the prognosis of patients.

### Ethical Approval Statement

This research was approved by the Ethics Committee of the Sun Yat-sen University Cancer Center, and informed consent was granted a waiver due to the retrospective nature of the study.

### Western Cohort

The data of GC patients from the US Surveillance, Epidemiology, and End Results (SEER) database, treated during the year 2004-2017, was retrieved using the SEER*Stat software (version 8.3.6). The database named “Incidence - SEER Research Data, 13 Registries, Nov 2019 Sub (1992-2017)”, was released in April 2019. Patients who had a “Site and Morphology. CS Schema - AJCC 6th Edition” data field of “Stomach” were selected using similar inclusion and exclusion criteria as the Chinese cohort. This Western dataset was used as the validation cohort to confirm the significance of the LNR stage and TRM staging system.

### The LNR Stage and TRM Staging System

The optimal LNR cutoff points were determined using the X-tile software (23) for classifying the LNR into five categories, namely LNR0, LNR1, LNR2, LNR3a, and LNR3b, to maintain consistency with the 8th AJCC nodal categories number (**Table 1**); based on which the novel TRM staging system was constructed. For the TRM staging system, the same T and M definitions as the 8th AJCC TNM staging system was used, while the R referred to the LNR categories.

**Table 1:** TRM/TNM staging system.

	N0/LNR0	N1/LNR1	N2/LNR2	N3a/LNR3a	N3b/LNR3b
T1	IA	IB	IIA	IIB	IIIB
T2	IB	IIA	IIB	IIIA	IIIB
T3	IIA	IIB	IIIA	IIIB	IIIC
T4a	IIB	IIIA	IIIA	IIIB	IIIC
T4b	IIIA	IIIB	IIIB	IIIC	IIIC

### Statistical Analysis

The Kaplan-Meier (KM) method was used to analyze patients’ OS. To assess the prognostic performance of the 8th AJCC TNM system, each TNM stage was stratified by TRM stages. In the same way, each TRM stage was stratified by TNM stages to assess the performance of the TRM staging system. The homogeneity across subgroups within each stage was compared using the log-rank test. Moreover, we used the likelihood ratio *x* ^2^ test within the Cox proportional hazard regression model to compare and evaluate the homogeneity of the TRM system and the TNM system. The Akaike information criterion (AIC) value and C-index value, related to a Cox regression model, were calculated for the two systems to measure discriminatory ability. A smaller AIC value and larger C-index value indicate a more desirable model for predicting outcome (24–27). Decision curve analyses, based on the Cox proportional-hazards model, were then applied to both the training set and validation set to compare performance of the TRM staging system and TNM staging system. Two-sided P values were calculated for all tests and a P value of less than 0.05 was considered statistically significant. Analyses were performed using the SPSS software (version 25.0) and R Studio (version 1.3.1093).

## Results

### Patients Characteristics

The data of 2,032 and 13,174 patients were retrieved from the SYSUCC and SEER databases, respectively. There were 697 female patients and 1,335 male patients in the training set, and 4,894 female patients and 8,280 male patients in the validation set, with a mean age of 57.4 ± 11.8 years (range 20-90 years) and 64.9 ± 12.8 years (range 20-85 years), respectively. The 5-year survival rates of the patients in training and validation set were 64.6% and 44.3%. The patients clinicopathological characteristics of both groups were listed in **Table 2**.

**Table 2:** Clinic-pathological Factors of training set and validation set.

Clinic-pathological Factors	Training set		Validation set	
	*n*	%	*n*	%
All	2032		13174	
Age				
≤39	183	9.0	363	2.8
40-59	903	44.4	3244	24.6
≥60	946	46.6	9567	72.6
Gender				
Male	1335	65.7	8280	52.9
Female	697	34.3	4894	37.1
Tumor size				NA
≤4cm	1009	49.7		
>4cm	987	48.6		
Unknown	36	1.8		
Histological grade				NA
Well	71	3.5		
Moderately	240	11.8		
Poorly/Undifferentiated	1652	81.3		
Unknown	69	3.4		
Gastrectomy Type				NA
Distal	1122	55.2		
Proximal	379	18.7		
Total	449	22.1		
Unknown	84	4.0		
No. of LNs				
≤15	330	16.2	6486	49.2
>15	1702	83.8	6688	50.8
pT stage				
T1	382	18.8	3394	25.8
T2	234	11.5	1770	13.4
T3	798	39.3	4357	33.1
T4a	539	26.5	2801	21.3
T4b	79	3.9	852	6.5
pN stage				
N0	643	31.6	6067	46.1
N1	318	15.6	2341	17.8
N2	356	17.5	2032	15.4
N3a	419	20.6	1779	13.5
N3b	296	14.6	955	7.2
LNR stage				
LNR0	643	31.6	6067	46.1
LNR1	348	17.1	1348	10.2
LNR2	360	17.7	1673	12.7
LNR3a	480	23.6	2146	16.3
LNR3b	201	9.9	1940	14.7

NA, Not Available.

### Comparison Between the 8th AJCC pN Stage and LNR Stage

**Figure 1:**
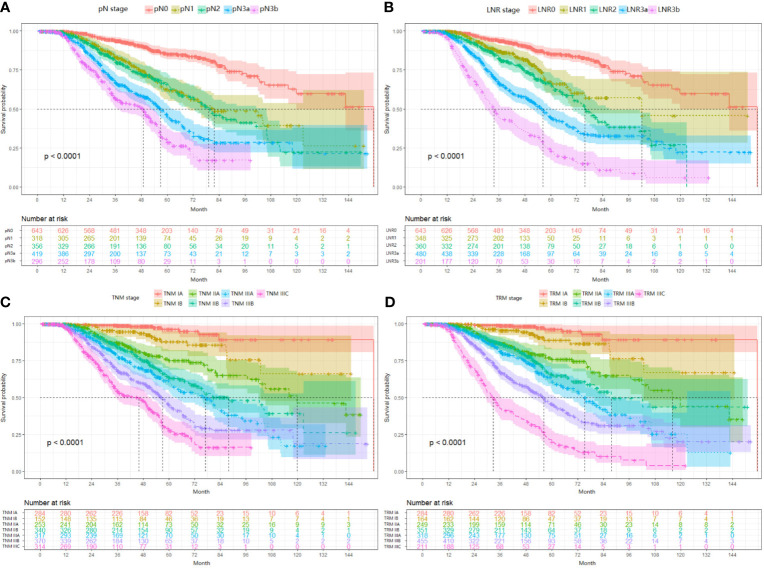
Impact of the pN **(A)**, LNR **(B)**, TNM **(C)**, and TRM **(D)** staging on OS in training set. **(A)** pN stage: pN0 vs pN1, *p* < 0.001; pN1 vs pN2, *p* = 0.546; pN2 *vs* pN3a, *p* < 0.001; pN3a *vs* pN3b, *p* < 0.001; **(B)** LNR stage: LNR0 *vs* LNR1, *p* < 0.001; LNR1 *vs* LNR2, *p* = 0.010; LNR2 *vs* LNR3a, *p* < 0.001; LNR3a *vs* LNR3b, *p* < 0.001; **(C)** TNM staging system: IA *vs* IB, P = 0.006; IB *vs* IIA, *p* = 0.004; IIA *vs* IIB, *p* = 0.016; IIB vs IIIA, *p* = 0.101; IIIA vs IIIB, *p* = 0.001; IIIB *vs* IIIC, *p* < 0.001; **(D)** TRM staging system: IA *vs* IB, *p* = 0.020; IB *vs* IIA, *p* = 0.001; IIA *vs* IIB, *p* = 0.185; IIB *vs* IIIA, *p* = 0.024; IIIA *vs* IIIB, *p* < 0.001; IIIB *vs* IIIC, *p* < 0.001. LNR, lymph node ratio; TNM, tumor–node–metastasis; TRM, tumor–ratio–metastasis.

For the training set, the 5-year OS for the pN stage categories (N0-N3b) was 86.5%, 67.0%, 62.9%, 45.7%, and 34.7% (P<0.001, log-rank test *x* ^2^ = 281.5, overall comparisons), respectively. Additionally, pairwise comparison showed significant difference (P<0.001) between-categories (pN0 vs pN1; pN2 vs pN3a; pN3a vs pN3b), except for pN1 vs pN2 (P=0.546) (**Figure 1A**).

For the LNR categories (LNR0-LNR3b), the 5-year OS was 86.5%, 74.6%, 62.6%, 45.1%, and 21.3% (P<0.001, log-rank test *x* ^2^ = 463.1, overall comparisons), respectively. Similar pairwise comparison showed significant difference (P<0.05) within each of the categories (**Figure 1B**). Then, the pN with LNR categories were compared. First, each pN category (N1–N3b) was stratified into LNR subgroups. Using the Kaplan-Meier method (log-rank test), significant heterogeneity in 5-year OS was found in each pN category (4/4, 100%). Second, each LNR category (LNR1-LNR3b) was also stratified into pN subgroups. Only significant heterogeneity in the 5-year OS of the LNR1 category was found (1/4, 25%) (**Table 3**).

**Table 3:** Five-Year OS by N Stage and LNR Stage for training set and validation set.

Training set	LNR stage					*p*
pN stage	LNR0	LNR1	LNR2	LNR3a	LNR3b	
N0	86.5 (*643*)*					NA
N1		68.7 (*246*)	68.3 (*54*)	38.5 (*13*)	20.0 (*5*)	<0.001
N2		92.1 (*98*)	60.2 (*169*)	43.9 (*76*)	35.2 (*13*)	<0.001
N3a		NA (*4*)	60.7 (*130*)	43.4 (*226*)	21.6 (*59*)	<0.001
N3b			51.4 (*7*)	45.6 (*165*)	19.7 (*124*)	<0.001
*P*	NA	0.004	0.422	0.408	0.408	
**Validation set**	**LNR stage**					***p***
**pN stage**	**LNR0**	**LNR1**	**LNR2**	**LNR3a**	**LNR3b**	
N0	62.8 (*6067*)					NA
N1		51.4 (*1185*)	33.0 (*740*)	24.0 (*231*)	6.3 (*185*)	<0.001
N2		47.4 (*155*)	39.0 (*773*)	26.5 (*850*)	17.5 (*254*)	<0.001
N3a		19.4 (*8*)	40.5 (*155*)	23.7 (*905*)	11.4 (*711*)	<0.001
N3b			0 (*5*)	22.3 (*160*)	9.4 (*790*)	<0.001
*P*	NA	0.262	0.028	0.864	0.061	

*5-year OS rate (%);(number of patients); NA, Not Available.

p<0.05 indicates significant heterogeneity between subgroups.

**Figure 2:**
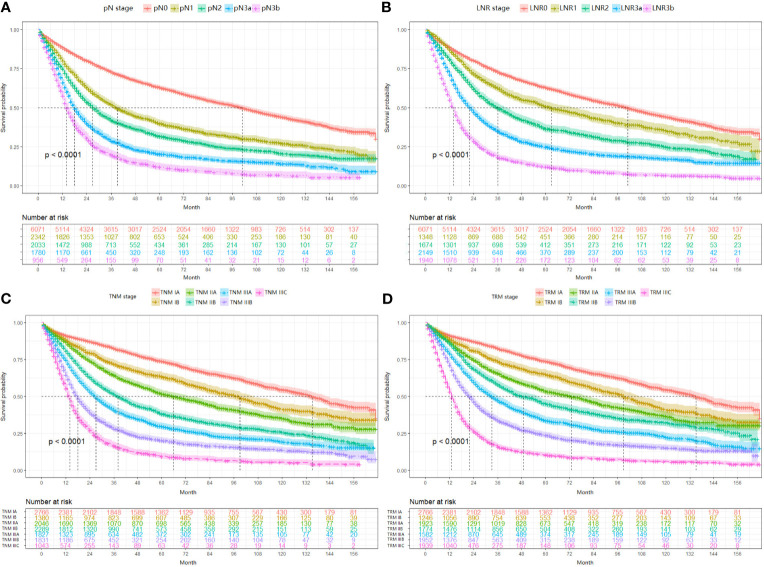
Impact of the pN **(A)**, LNR **(B)**, TNM **(C)**, and TRM **(D)** staging on OS in validation set. **(A)** pN stage: pN0 *vs* pN1, *p* < 0.001; pN1 *vs* pN2, *p* < 0.001; pN2 *vs* pN3a, *p* < 0.001; pN3a *vs* pN3b, *p* < 0.001; **(B)** LNR stage: LNR0 *vs* LNR1, *p* < 0.001; LNR1 *vs* LNR2, *p* < 0.001; LNR2 *vs* LNR3a, *p* < 0.001; LNR3a *vs* LNR3b, *p* < 0.001; **(C)** TNM staging system: IA *vs* IB, *p* < 0.001; IB *vs* IIA, *p* < 0.001; IIA *vs* IIB, *p* < 0.001; IIB *vs* IIIA, *p* < 0.001; IIIA *vs* IIIB, *p* < 0.001; IIIB *vs* IIIC, *p* < 0.001; **(D)** TRM staging system: IA *vs* IB, *p* < 0.001; IB *vs* IIA, *p* < 0.001; IIA *vs* IIB, *p* < 0.001; IIB *vs* IIIA, *p* < 0.001; IIIA *vs* IIIB, *p* < 0.001; IIIB *vs* IIIC, *p* < 0.001. LNR, lymph node ratio; TNM, tumor–node–metastasis; TRM, tumor–ratio–metastasis.

For the validation set, 5-year OS for the pN stage categories (N0-N3b) was 62.8%, 39.7%, 31.6%, 20.2%, and 11.7% (P<0.001, log-rank test *x^2^* = 2240.8, overall comparisons), respectively. For the LNR categories (LNR0-LNR3b), the 5-year OS was 62.8%,50.8%, 36.4%, 24.8%, and 11.9% (P<0.001, log-rank test *x* ^2^ = 2880.8, overall comparisons), respectively. Pairwise comparison showed that both the pN and the LNR category had a good discriminatory ability among each category (**Figures 2A, B**). Then after stratified analyses, significantly heterogeneous 5-year OS was observed in each of the pN category (4/4, 100%), howreve, significantly heterogeneous 5-year OS was only found in LNR2 category (1/4, 25%) (**Table 3**).

### Comparison Between the 8th AJCC TNM System and Hypothetical TRM System

First, since the TRM IA stage is equal to TNM IA stage, each TRM stage (except IA) was stratified into TNM stage subgroups to estimate the performance of the novel TRM system. Second, the homogeneity of the 5-year OS for subgroups within every TRM stage was evaluated using the log-rank test. Finally, a same stratified analysis method was performed in each TNM stage (except IA). For the training set, we found that only 2 (IIA, IIB) of the 6 TRM subgroups showed statistically heterogeneous 5-year OS, while up to 5 of 6 TNM stage subgroups (except IIA) had statistically heterogeneous 5-year OS (**Table 4**). The K-M plots of TNM stage and TRM stage were shown in **Figures 1C, D**. In the validation set, we used the same stratified analyses between the two systems. There was only 1 of 6 TRM stage subgroups (IIIC) showed statistically heterogeneous 5-year OS, while all of the 6 TNM stage subgroups showed statistically heterogeneous (**Table 4**). The K-M plots of TNM stage and TRM stage were shown in **Figures 2C, D**.

**Table 4:** Five-Year OS by TNM Stage and TRM Stage for training set and validation set.

Training set	TRM							*p*
TNM	IA	IB	IIA	IIB	IIIA	IIIB	IIIC	
IA	96.7 (*284*)*							NA
IB		91.1 (*150*)	50.0 (2)					<0.001
IIA		NA (*13*)	76.3 (*227*)	73.8 (*13*)				0.360
IIB		NA (*1*)	NA (*20*)	65.3 (*253*)	71.4 (*47*)	40.0 (*15*)	25.0 (*4*)	<0.001
IIIA				89.2 (*77*)	54.8 (*155*)	41.6 (*74*)	31.8 (*11*)	<0.001
IIIB				NA (*6*)	57.2 (*109*)	42.3 (*208*)	18.0 (*47*)	<0.001
IIIC					51.4 (*7*)	45.2 (*158*)	19.0 (*149*)	<0.001
*P*	NA	0.661	<0.001	0.020	0.323	0.470	0.608	
**Validation set**	**TRM**							***p***
**TNM**	**IA**	**IB**	**IIA**	**IIB**	**IIIA**	**IIIB**	**IIIC**
IA	73.8 (*2766*)							NA
IB		65.2 (*1231*)	50.0 (*110*)	60.0 (*25*)	35.7 (*14*)			<0.001
IIA		50.9 (*15*)	53.7 (*1782*)	41.3 (*184*)	25.0 (*45*)	45.9 (*20*)		<0.001
IIB			53.1 (*30*)	45.3 (*1447*)	30.3 (*538*)	20.4 (*165*)	12.2 (*109*)	<0.001
IIIA			NA (*1*)	47.6 (*112*)	35.0 (*843*)	23.5 (*679*)	17.7 (*192*)	<0.001
IIIB				22.2 (*6*)	40.4 (*137*)	24.1 (*943*)	11.5 (*745*)	<0.001
IIIC					40.0 (*5*)	22.8 (*145*)	7.1 (*893*)	<0.001
*P*	NA	0.215	0.519	0.255	0.230	0.185	0.005	

*5-year OS rate (%);(number of patients); NA, Not Available.

p<0.05 indicates significant heterogeneity between subgroups.

### Comparison Between the Two Systems Within the Patients With Over 15 LNs Retrieved in Validation Set

For patients with more than 15 examined lymph nodes, the TRM staging system was also superior to TNM staging system. The log-rank *x* ^2^ of LNR stage (*x* ^2^ = 1723.7) was larger than that of pN stage (*x* ^2^ = 1552.3). For the stratified analyses, every pN stage (except pN0) showed significantly heterogeneous 5-year OS (pN1: P=0.004; pN2-pN3b: P<0.001) by stratifying into LNR subgroups, but only LNR3b showed significantly heterogeneous results(LNR3b: P=0.018; LNR1: P=0.064; LNR2: P=0.953; LNR3a: P=0.654). Additionally, 5 of 6 TNM stages (except IIB) showed significantly heterogeneous by stratified into TRM subgroups (TNM IB: P=0.014; TNM IIA: P=0.009; TNM IIB: P=0.092; TNM IIAB: P= 0.001; TNM IIIB/IIIC: P<0.001), while only TRM IIIC showed significantly heterogeneous results (TRM IB: P=0.065; TRM IIA: P=0.513; TRM IIB: P=0.194; TRM IIAB: P= 0.706; TNM IIIB: P=0.749; TRM IIIC: P<0.001). Similar results were observed for the patients with over 15 LNs retrieved in the training set. Due to space limitations, there is no further description here.

### Compared the Performance Between the TRM and TNM Systems

**Figure 3:**
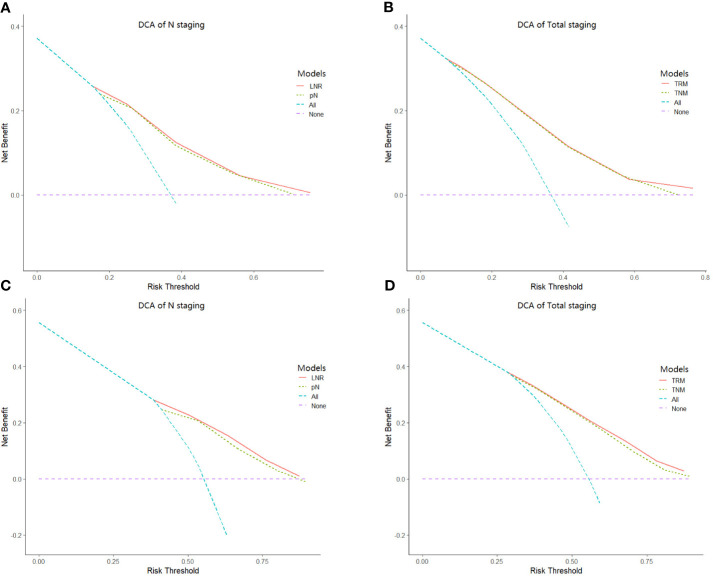
Results of decision curve analysis. Results of decision curve analysis pN *vs* LNR **(A)** and TNM *vs* TRM **(B)** in the training set; pN vs LNR **(C)** and TNM *vs* TRM **(D)** in the validation set.

In this part, the performance between the TRM staging system and TNM staging system, including the comparison between LNR and pN stage, was evaluated by the likelihood ratio *x* ^2^, linear trend *x* ^2^, C-index, and AIC value (**Table 5**). For both the training set and validation set, the TRM staging system showed better homogeneity (larger likelihood ratio *x*^2^), discriminatory ability, and monotonicity of gradients (larger linear trend *x* ^2^) than the TNM staging system. Moreover, the C-index of the TRM staging system was larger than that of the TNM staging system, while the AIC value of the TRM staging system was smaller than the TNM staging system. The results of the C-index and AIC value also indicate that the performance of the TRM system is better than the TNM system. The results of decision curve analyses comparing the performance between the TRM and TNM systems are shown in **Figure 3**, which indicate that both the LNR stage and TRM stage have greater net benefit than the pN stage and TNM stage, both in the training set and validation set.

**Table 5:** Comparison of the performance of the AJCC TNM staging system and TRM staging system.

Group	Classification	Subgroups	Likelihood ratio *x* ^2^	Linear trend *x* ^2^	C-index	AIC value
Training set	pN stage	N0-N3b	281.41	185.39	0.69	8219.14
	LNR stage	LNR0-LNR3b	462.92	291.99	0.72	8156.79
	TNM stage	IA-IIIC	340.42	248.16	0.71	8130.17
	TRM stage	IA-IIIC	534.39	332.32	0.73	8075.77
Validation set (Total)	pN stage	N0-N3b	2202.81	1579.91	0.65	132895.23
	LNR stage	LNR0-LNR3b	2831.63	1946.94	0.66	132447.64
	TNM stage	IA-IIIC	2768.89	2100.87	0.67	132301.03
	TRM stage	IA-IIIC	3353.71	2347.63	0.68	131988.44
Validation set (> 15 LNs)	pN stage	N0-N3b	1526.89	1100.26	0.68	57880.95
	LNR stage	LNR0-LNR3b	1695.30	1158.17	0.68	57782.85
	TNM stage	IA-IIIC	1762.64	1235.62	0.69	57701.60
	TRM stage	IA-IIIC	1908.22	1281.39	0.70	57624.00

## Discussion

In the present study, we constructed a novel LNR stage as an alternative to pN stage, and replaced pN of the TNM staging system with LNR so as to set up the TRM staging system. Our results indicate that both the LNR stage and TRM staging system could bring more accurate patients stratification and more prognostic value compared with the pN stage and TNM staging system (AJCC 8th edition).

In 2002, metastatic lymph node ratio (MLR) was put forward and used to predict the prognosis of GC patients for the first time (17). After that, several studies have confirmed that staging by the LNR rather than absolute number of MLN could better predict prognosis of gastric cancer patients. For instance, Xiao et al. (28) analyzed the prognosis of 1,042 GC patients who underwent D2 gastrectomy with less than 16 retrieved lymph nodes and divided LNR into four subgroups (0%, 1%-30%, 31%-50%, >50%). They demonstrated that using LNR stage could predict the prognosis better than pN stage when the number of lymph nodes examined was less than 15. Lee et al (29) analyzed 3284 patients from eight institutions and categorized LNR into five subgroups (0, 0-0.06, 0.06-0.27, 0.27-0.49, and >0.49). They demonstrated that LNR stage was a better predictor of prognosis for GC patients than the pN stage. Wang et al. (13) analyzed 18,043 gastric cancer patients from SEER database and classified LNR into five subgroups (0, 0<LNR ≤ 1/15, 1/15<LNR ≤ 3/10, 3/10<LNR ≤ 7/10 and LNR>7/10). They found that using the TRM system caused less misclassification than the TNM system (12% *vs* 57%).

However, the previous studies had several limitations: (i) The sample size included in the study was relatively small (a few hundred to several thousands) or only from a single institution. (ii) Most previous studies drew their conclusion based on old versions of AJCC TNM edition rather than the new version. (iii) Most previous studies only compared the prognosis value of LNR and pN staging. They did not combine the LNR stage and pT stage to construct a hypothetical TRM staging system. (iv) Most previous studies determined the cut points of LNR by using the so-called ‘best cut-off method by log-rank test’, ignoring the fact that LNR should be a continuous variable.

In the present study, we avoided the limitations mentioned above. We analyzed 15,206 GC patients from the Sun Yat-sen University Cancer Center (Training set; n=2,032) and the US Surveillance, Epidemiology, and End Results (SEER) database (Validation set; n=13,174). We did not neglect the differences in the multimodal treatment of gastric cancer between East and West (30–32). Furthermore, we fully considered the LNR as a continuous variable, and we used the X-tile software to determine the optimal cut-off point. And we demonstrated that TRM staging system showed the superiority to the 8th AJCC TNM staging system for the reasons as follows: (i) In univariate analysis, the log-rank *x* ^2^ of LNR stage (training set: 463.1; validation set: 2880.8) was larger than that of pN stage (training set: 281.5; validation set: 2240.8), which indicated a higher statistical significance. Stratified analyses using the Kaplan-Meier method identified significantly heterogeneous OS in every pN category but only one LNR category (**Table 3**). (ii) In univariate analysis, the log-rank *x*^2^ of TRM stage (training set: 534.58; validation set: 3412.03) was larger than that of TNM stage (training set: 340.54; validation set: 2816.64), which indicated a higher statistical significance. Stratified analyses identified significantly heterogeneous OS in almost every TNM stage but only one TRM stage (**Table 4**). (iii) For patients with > 15 lymph nodes examined in the validation set, stratified analyses showed that five of the six TNM stages (except IIB) showed significantly heterogeneous results, while only TRM IIIC showed significantly heterogeneous results.

Based on Ueno et al (27), regarding the discriminatory ability between different groups, the monotonicity of gradients reflected in the relationship between stages and OS, and the homogeneity within subgroups, a model with larger linear trend *x* ^2^ and the likelihood ratio *x* ^2^ was considered as the better model. In our study, the TRM staging system had better discriminatory ability, monotonicity of gradients (larger liner trend *x*^2^), and homogeneity (larger likelihood ratio *x*^2^). Furthermore, the TRM staging system with a smaller AIC value and a lager C-index value indicated the better prognostic stratification. These results confirmed that the TRM staging system could predict the prognosis of GC patients better than the TNM staging system (**Table 5**). And moreover, the results of decision curve analyses indicate that the TRM staging system has a greater net benefit than the TNM staging system. Although our study demonstrated the superiority of the TRM staging system, it does not mean the surgeons could perform a lesser extent of lymph node dissection. Actually, retrieving as many as possible lymph nodes could predict the prognosis of patients better (33).

There were several limitations in this study. First, as a retrospective study, some data were inevitably missing or not applicable for analysis. For instance, we did not include data on the adjuvant chemotherapy in our study. If postoperative treatments varied greatly between patients, that could bring confounding effects to the study. However, as patients in the training set were from a single cancer center, and physicians would normally prescribe 5-fluorouracil (5-FU) based recipe for those TNM IIA or higher staged GC patients according to latest GC guidelines, the postoperative treatments for our patients were relatively homogeneous. So we did not include this data in the present. Second, our TRM staging system was similar to the TNM staging system. We just replaced the pN stage with the LNR stage, based on which the novel TRM staging system was constructed. Actually, we could improve our TRM staging system with the better combination of the LNR stage and pT stage, but we think that the staging system will include additional non-anatomical factors in the future.

## Conclusion

Our findings showed that the LNR and TRM staging systems could better estimate the 5-year OS than the 8th edition AJCC pN staging and TNM staging system, due to less stage migration. However, a more extensive lymph node dissection and to standardize the LNR cutoff value is still needed to extend and use LNR/TRM staging system.

## Data Availability Statement

The raw data supporting the conclusions of this article will be made available by the authors, without undue reservation.

## Ethics Statement

The studies involving human participants were reviewed and approved by Ethics Committee of the Sun Yat-Sen University Cancer Center. Written informed consent for participation was not required for this study in accordance with the national legislation and the institutional requirements.

## Author Contributions

All authors took part in conception and design of this work, acquisition and collection of data, analysis of data, and participated in writing and reviewing this manuscript. All authors contributed to the article and approved the submitted version.

## Funding

This project was supported by 1. Chinese Society of Clinical Oncology (Roche foundation grants No: Y-2019Roche-157), 2. Science and Technology Project of Guangdong (grant No. 2014A020212331) and 3. National Natural Science Foundation of China (grant No.82001672).

## Conflict of Interest

The authors declare that the research was conducted in the absence of any commercial or financial relationships that could be construed as a potential conflict of interest.

The RE ZX declared a shared affiliation with the authors to the handling editor at time of review.
